# Recurrent Struma Ovarii Presented with High Levels of Thyroglobulin

**DOI:** 10.1155/2021/8868095

**Published:** 2021-03-22

**Authors:** Christiana Oikonomou, Nikol Spathari, Stavroula Doumoulaki, Antonella Koutela, Christos Stagkoglou, Dimitris Keramidaris

**Affiliations:** ^1^Fifth Colorectal Surgical Department, Metropolitan General Hospital, Athens, Greece; ^2^Private Sector, Athens, Greece; ^3^417 NIMTS, Veteran Hospital, Athens, Greece

## Abstract

Struma ovarii are rare ovarian tumors, of monodermal germ cell origin, containing predominantly thyroid tissue. They are typically benign unilateral pelvic masses. Among the rare cases of malignancy, the most common histological type is that of the papillary carcinoma. A definite preoperative diagnosis of these tumors is difficult to achieve since most imaging findings are generally nonspecific. A rare exception is the case of synchronous abnormal thyroid blood tests where an ovarian teratoma should be highly suspected, especially when no pathology of the thyroid gland coexists. Surgical excision is the mainstay of treatment. Taking into account that struma ovarii are mainly encountered in patients of a childbearing age, a conservative surgical approach is a reasonable option. We present the case of a 56-year-old woman who was diagnosed with both a primary and recurrent struma ovarii while investigating the incidental finding of elevated blood laboratory levels of the thyroglobulin (Tg).

## 1. Introduction

Ovarian teratomas are germ cell tumors encountered mainly in women of reproductive age and account for 10-20% of all ovarian neoplasms [1]. They usually contain variable tissues derived from different germ layers like hair, teeth, bone, muscle, adipose tissue, and thyroid tissue. The struma ovarii is a monodermal variant of teratomas often characterized as an ovarian goiter that contains predominantly thyroid tissue [2]. This pathological entity is usually benign. Malignancy is rarely diagnosed mostly in patients in their 6^th^ or 7^th^ decade of life, and papillary carcinoma is the most common histological type [3].

Clinical and imaging findings of these tumors are generally nonspecific; therefore, the diagnostic accuracy in the preoperative basis is difficult to achieve. However, synchronous abnormal blood laboratory levels of the thyroid function may raise the suspicion of an ovarian goiter, especially when no pathology of the thyroid gland is described.

## 2. Case Presentation

We present the rare case of an otherwise healthy 56-year-old woman who was initially referred to our department due to incidentally found high levels of serum thyroglobulin (Tg) (2500 ng/ml, normal values: 1.4–78). The levels of the thyroid hormones (T_3_, T_4_), the thyroid-stimulating hormone (TSH), and the antithyroid antibodies were within normal range values. A neck ultrasonography (US) had already been performed describing a heterogeneous thyroid gland and bilateral small nodules with benign radiological patterns.

A magnetic resonance imaging (MRI) of the neck was not diagnostic demonstrating no remarkable pathological findings of the thyroid gland.

**Figure 1:**
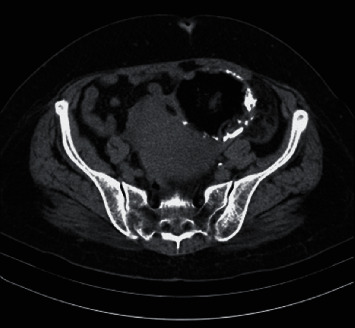
Preoperative CT scan.

Further investigation was followed to exclude an ectopic origin of Tg. A US of the abdomen followed by a computed tomography scan (CT) of the upper and lower abdomen was performed. A heterogeneous mass sized 11 × 9 × 9.9 cm was detected in the pelvis. The lesion had irregular cystic and solid components, findings highly suggestive of an ovarian teratoma. The tumor was contiguous with the left ovary with no signs of invasion to the neighbor organs. The right ovary showed no pathology, and the uterus had features of fibromatosis. No other intraabdominal pathology was described (Figure 1).

**Figure 2:**
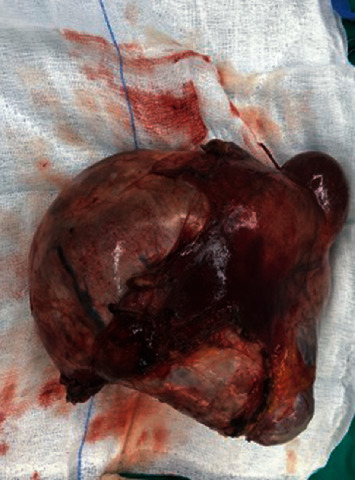
Ovarian teratoma of the left ovary.

Taking into consideration these findings, surgical excision of the adnexal mass was the treatment of choice. Provided that Tg levels remained high postoperatively, the option of a complementary total thyroidectomy was also discussed with the patient. Given the patient's postmenopausal age, a laparoscopic total hysterectomy with bilateral salpingo-oophorectomy was initially proposed. However, taking into account the patient's preference as well as the fact that no pathology of the right ovary was detected, a more conservative approach was finally decided. A laparoscopic left salpingo-oophorectomy en bloc with the irregular mass was performed. The tumor was removed intact, preserving its capsule and preventing any content spillage (Figure 2). The patient's postoperative course was uneventful.

Macroscopic examination of the tumor showed an irregular grey-yellow cystic neoplasm, filled with hair and fat-like tissue. Histopathological report set the diagnosis of a left ovarian mature cystic teratoma composed predominantly of thyroid tissue. Thyroid follicles were revealed in fibrous septa, and the immunohistochemistry result was positive for thyroglobulin. No remarkable findings were observed in the left fallopian tube.

During a three-month follow-up period, the Tg blood levels were within the normal range. An additional US of the thyroid gland confirmed no remarkable findings. In accordance with the patient's desire, we recommended an annual follow-up including laboratory and imaging examinations avoiding any further surgical procedure.

**Figure 3:**
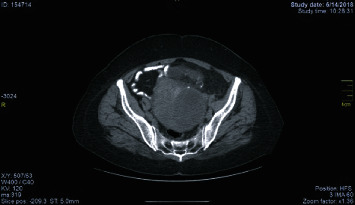
CT scan of the recurrent struma ovarii.

Six months later, the patient readmitted in our department due to a new remarkable increase in the Tg blood levels far above the normal (>1500 ng/ml). A CT scan of the lower abdomen demonstrated a 11 × 10.8 × 11.5 cm pelvic mass. The lesion was located centrally in the pelvis and consisted of solid and cystic compartments. Additionally, a limited fluid collection was also revealed in the right pelvis. No other pathology was detected (Figure 3).

An early recurrence of the struma ovarii was suspected. Following written informed consent from the patient, an open total hysterectomy with right salpingo-oophorectomy was successfully performed. The pelvic mass was removed en bloc with the remaining genital organs. Neither rupture of the tumor nor intraperitoneal seeding was encountered. The patient had an uncomplicated postoperative period.

The pathologic report described a grey-yellow mass sized 12 × 8.5 × 7.4 cm totally occupying the right ovary. A nodular lesion with a maximal diameter of 0.4 cm was additionally detected at the uterine wall. The microscopic examination reported typical findings of an ovarian teratoma. Immunohistochemical markers of the thyroid tissue were found positive, and the definitive diagnosis of a benign struma ovarii was set. The right fallopian tube had a normal configuration and histology. Lastly, the nodular lesion of the uterus was found to be a small leiomyoma.

During a one-year follow-up period, the patient is in a good clinical condition. Tg levels remain within the normal range, and consecutive US examination of the thyroid gland as well as of the lower abdomen has not revealed any pathology.

## 3. Discussion

Stroma ovarii are rare tumors diagnosed typically in women between the ages of 40 and 60 years old. They account for nearly 5% of all the ovarian tumors [1]. These types of neoplasms are defined as ovarian teratomas that consist mostly of thyroid tissue, in a proportion of more than 50% of the overall mass. They represent the most common type of monodermal teratomas. The majority of these neoplasms are benign, and only 5% of all the reported cases are malignant, mainly follicular or papillary thyroid carcinoma [2].

Most stroma ovarii are asymptomatic and are detected incidentally during a routine medical examination. In some reports, these tumors are characterized as slowly enlarging pelvic masses associated with abdominal pain or other nonspecific symptoms [3]. As regards the function of the thyroid gland, in most patients, the thyroid hormone levels are within the normal range. Hyperthyroidism has been reported in 5%-8% of the cases, possibly as a result of TSH receptor antibodies or autonomous thyroid hormone secretion [2, 4]. According to recent literature, stroma ovarii are the only neoplasms reported to produce Tg [5].

In our case, the high levels of Tg in combination with a grossly normal thyroid gland raised the suspicion of an ectopic origin of the Tg.

Generally, mature teratomas have characteristic features in the US and CT imaging. A typical image in the US shows a heterogenous adnexal mass with smooth contours and acoustic shadowing due to sebaceous material or/and thin echogenic bands due to hair follicles inside the cystic cavity [6]. A characteristic CT image demonstrates a multicystic tumor or mixed solid and cystic lesions with areas of low attenuation representing fat tissue as well as calcified areas representing either tissue calcification or dental components of the mass [7]. The cyst wall is not typically enhanced after intravenous administration of contrast material. MRI is a highly sensitive modality that can accurately describe the benign or malignant nature of the tumor. MRI findings are more specific, with variable signal intensity in T1- and T2- weighted images, depending on the content of the cyst. Low T1 and T2 signal shows simple cysts, whereas intermediate T1 and low T2 signal demonstrates colloid-filled follicles, resembling thyroid microscopic appearance [1, 8]. Fat suppression techniques are used in MRI to evaluate the fat component of the tumor [9].

Regardless of the accuracy of the imaging modalities, no definite preoperative diagnosis can be made. Therefore, it is the histopathological report of the tumor that sets a definite diagnosis [6].

The necessity of a second pathologoanatomical opinion is highlighted in the literature, given the rarity of these cases [5]. Immunohistochemistry and immunocytochemistry tests are an important guide for the correct diagnosis. Stroma ovarii generally have a positive immunostaining for Tg, as in our case. Complementary detection for RET/PTC rearrangements and BRAF mutations from the tissue sample is highlighted in other reports to examine the malignant potential of the tumor [10].

Sufficient evidence regarding the management of stroma ovarii supports that surgical excision is the treatment of choice. There are very few data available comparing radical versus conservative surgical approach and no established guidelines regarding the extensiveness of the surgical procedure. The option of a more conservative approach is supported in most case series, especially when childbearing women are involved and taking into consideration that struma ovarii arise mostly unilaterally, usually from the left ovary [3].

A similar approach is as well recommended even when there is a high index of suspicion for malignancy, mainly due to the low metastatic potential of the tumor and low risk of recurrence. In contrast, total hysterectomy with bilateral salpingo-oophorectomy or/and omentectomy can be a reasonable choice for postmenopausal women or those without maternity plans [11]. Metastatic disease in the abdominal cavity has been rarely reported in the fallopian tubes, the contralateral ovary, the para-aortic lymph nodes, the greater omentum, and pelvic walls [12]. Distant metastases are even less common, accounting for less than 5% of the cases [3].

In this regard, lymph node dissection in the para-aortic lymph nodes is mostly advocated when a mucinous variant of the struma ovarii is diagnosed due to their lymphatic pattern of spread [13]. In case of metastatic disease, some studies support a more aggressive strategy including total thyroidectomy and Iodine-131 ablation of any thyroid residues as well as metastatic lesions [2, 14]. In such cases, the histopathology of the thyroid gland is an important feature for differentiation between a metastatic thyroid cancer and intra-abdominal involvement from a malignant ovarian tumor [3].

In the presented case, our initial approach was oriented towards a more conservative surgical procedure, in accordance with the patient's decision. However, in the event of a recurrent tumor, a more extensive surgical procedure was performed in an attempt to eliminate the risk of recurrence. The option of a complementary total thyroidectomy was finally avoided since the laboratory levels of Tg remained low and no remarkable pathology of the thyroid gland was detected during consecutive diagnostic imaging.

## 4. Conclusion

Struma ovarii are rare and mainly asymptomatic ovarian tumors making preoperative diagnosis quite challenging. Physicians should maintain an appropriately high index of suspicion for an ovarian goiter, mostly in cases of female patients presented with elevated laboratory levels of the Tg and no synchronous pathology of the thyroid gland. Additionally, postoperative increase in Tg levels can be a useful indicator of recurrence during the follow-up period.

## Data Availability

Data are deposited in a repository.
